# Anti-spike antibody level is associated with the risk of clinical progression among subjects hospitalized with COVID-19 pneumonia: results from a retrospective cohort study

**DOI:** 10.1007/s15010-024-02250-9

**Published:** 2024-04-23

**Authors:** Giuseppe Lapadula, Luca Mezzadri, Giustina Lo Cascio, Laura Antolini, Sergio Malandrin, Alice Ranzani, Silvia Limonta, Annalisa Cavallero, Paolo Bonfanti

**Affiliations:** 1 Infectious Diseases Unit, Fondazione IRCCS San Gerardo dei Tintori, Monza, Italy; 2 School of Medicine and Surgery, University of Milano-Bicocca, Milan, Italy; 3 Bicocca Bioinformatics Biostatistics and Bioimaging Center-B4, University of Milano-Bicocca, Milan, Italy; 4 Microbiology Unit, Fondazione IRCCS San Gerardo Dei Tintori, Monza, Italy

**Keywords:** COVID-19 pneumonia, Anti-Spike antibody level, SARS-CoV-2, Vaccination, Clinical progression

## Abstract

**Purpose:**

Antibodies against SARS-CoV-2 spike (anti-S) may confer protection against symptomatic COVID-19. Whether their level predicts progression among those with COVID-19 pneumonia remains unclear.

**Methods:**

We conducted a retrospective cohort study to assess predictors of anti-S levels and whether anti-S titer is associated with death or mechanical ventilation (MV). Adults hospitalized for COVID-19 pneumonia between July 2021 and July 2022 were enrolled if anti-S had been measured within 72 h of admission. Predictors of anti-S level were explored using multivariable quantile regression. The association between anti-S levels and 30-day death/MV was investigated via multivariable logistic regression. Analyses were stratified by vaccine status.

**Results:**

The median anti-S level was 1370 BAU/ml in 328 vaccinated and 15.5 BAU/ml in 206 unvaccinated individuals. Among the vaccinated, shorter symptom duration (*p* = 0.001), hematological malignancies (*p* = 0.002), and immunosuppressive therapy (*p* = 0.004) were associated with lower anti-S levels. In the unvaccinated group, symptom duration was the only predictor of anti-S levels (*p* < 0.001). After 30 days, 134 patients experienced death or MV. Among vaccinated individuals, higher anti-S levels correlated significantly with lower death/MV risk (per log_2_ increase, OR 0.88, 95%CI 0.81–0.97), irrespective of age and solid malignancies. Among unvaccinated, a marginally protective effect was observed (OR 0.86, 95%CI 0.73–1.01), independent of age, immunosuppressive therapy, and diabetes. Adjustment for monoclonal antibody treatment strengthened the association (OR 0.81, 95%CI 0.68–0.96).

**Conclusion:**

This study suggests that levels of anti-S antibodies can predict critical or fatal outcomes in COVID-19 pneumonia patients, regardless of vaccination. Whether anti-S Ab could guide risk assessment and vaccination boosting merits further evaluation.

## Background

The clinical presentation of coronavirus disease 2019 (COVID-19) varies greatly from patient to patient, ranging from asymptomatic to severe illness [1]. Though factors influencing progression to severe disease are not fully understood, studies showed that a robust and timely immune response against Severe Acute Respiratory Syndrome Coronavirus 2 (SARS-CoV-2) can avert severe manifestations of COVID-19. In particular, the production of antibodies that target the spike protein of SARS-CoV-2 (anti-S) plays a critical role in the immune response [2] and holds promise as a potential prognostic marker [3, 4].

Prior research has indicated that a delayed or impaired antibody response during the early stages of natural infection can lead to fatal outcomes [5–7]. Nonetheless, other reports have suggested that a rapid development of antibodies alone does not guarantee protection against death [8]. Latest studies on vaccinated individuals or convalescent cohorts have also shown that increasing levels of neutralizing or binding antibodies are linked to protection from symptomatic and severe COVID-19 [3, 9–14]. On the other hand, however, detectable anti-S antibodies do not confer complete protection from breakthrough infections [12, 15]. Moreover, mortality rates among those who develop severe disease despite vaccination remain substantial [16–18]. As a result, it is yet to be determined whether the measurement of antibody level can serve as a prognostic marker for the progression to critical illness or death in patients with COVID-19-associated pneumonia.

Therefore, in the present study, we aimed at assessing what factors are associated with a more robust humoral response among hospitalized patients with COVID-19-related pneumonia and whether, in these patients, anti-S antibody levels can help predict the risk of disease progression to critical respiratory failure or death.

## Materials and methods

### Study design

We conducted an observational retrospective cohort study enrolling all consecutive patients admitted for COVID-19 pneumonia between July 1st 2021 and July 31st 2022 in the “Fondazione IRCCS San Gerardo dei Tintori” Hospital, a tertiary referral academic hospital, located in Monza, Lombardy, Northern Italy.

Patients were eligible if: ≥ 18 years old, had a positive nasopharyngeal swab for SARS-CoV-2 RNA upon admission and had radiological or clinical evidence of COVID-19-related pneumonia, defined as peripheral oxygen saturation (SpO_2_) < 94% on room air or need for oxygen therapy. In addition, anti-S antibody determination within 72 h of hospital admission had to be available. Patients hospitalized for reasons different from COVID-19, who incidentally tested positive for SARS-CoV-2 at admission, were excluded. Information on patient characteristics and disease course was obtained from the hospital electronic medical records. The following information was collected: age, gender, vaccine status, symptom duration, presence of immunosuppressive comorbidities (hematological malignancy, solid tumor, grade ≥ 4 chronic kidney disease or end-stage renal disease, diabetes, liver cirrhosis, HIV, organ transplant, use of immunosuppressive drugs), variant of infecting SARS-CoV-2 (where available), disease clinical severity at admission, treatment with the monoclonal antibodies (mAb) casirivimab/imdevimab or tixagevimab/cilgavimab, need for mechanical ventilation during hospitalization, discharge date, vital status, and conditions at discharge.

### Anti-spike antibody determination

Anti-S levels were measured using LIAISON® SARS-CoV-2 TrimericS IgG assay, a chemiluminescence immunoassay developed by Diasorin S.p.A (Saluggia, Italy) for the quantitative determination of IgG against the viral spike protein in the native trimeric conformation. This assay targets two critical regions within the S1 subunit of the spike protein: the receptor-binding domain (RBD), responsible for binding to the human angiotensin-converting enzyme receptor-2 and recognized as highly immunogenic, and the N-Terminal Domain, located apart from the RBD. The results of the quantitative determination of specific IgG antibodies were expressed in BAU/ml (binding antibody unit). The assay's measurement range spanned from 4.81 to 2080 BAU/mL [19]. Previous studies showed that the LIAISON® assay strongly correlates with virus neutralizing assays, which represent the gold standard methods for assessing anti SARS-CoV-2 antibodies but whose practical application in clinical settings is limited due to technical requirements [20, 21].

### Statistical analysis

We explored possible predictors of anti-S level using quantile regression over the median value. The following covariates, deemed to be possibly associated with humoral response, were explored: age, gender, symptom duration, clinical severity of COVID-19 and presence of the above-mentioned immunosuppressive conditions. In order to discern the distinct impacts of vaccination on antibody responses and to account for baseline differences in immunity and potential predictors, separate analyses were conducted for vaccinated and unvaccinated individuals. Therefore, two separate multivariable models were constructed, selected to maximize model fit, and guided by clinical reasoning.

The time to the occurrence of a composite outcome of death or need for mechanical ventilation was visually depicted by estimating survival probabilities via the Kaplan–Meier method. Patients within both the vaccinated and unvaccinated cohorts were compared across age strata and antibody titer levels, the latter classified into tertiles, using the log-rank statistics.

The association between anti-S level and the risk of 30-day clinical progression, defined as death or need for mechanical ventilation, was evaluated using uni- and multivariable logistic regression models. To determine the best functional form of the anti-S titer as the explanatory variable, several models were explored (linear effect, cubic spline, quadratic, linear effect on logarithm). The model using log_2_ transformed anti-S titer had the lowest prediction error, as measured by Brier score, and was therefore selected. The following covariates, considered to be potential confounders of the association between anti-S titer and the study outcome, were explored for possible inclusion in the final multivariable models: age, gender, days since symptoms’ onset, disease clinical severity, presence of hematological malignancy, solid tumor, grade ≥ 4 chronic kidney disease or end-stage renal disease, diabetes, liver cirrhosis, HIV infection, organ transplant or use of immunosuppressive drug. As previously explained, separate models were constructed for vaccinated and unvaccinated individuals. Among vaccinated individuals the predicted probability of 30-day clinical progression was estimated as function of the log_2_ transformed anti-S titer and represented graphically. This enables to identify a cut-point with corresponding predicted probability equal to the observed probability of 30-day clinical progression among vaccinated. Multivariable models presented in this paper are considered the most clinically and statistically significant.

Because treatment with mAb was suspected to act as a mediator of the relationship between anti-S titer and the outcome of interest among unvaccinated subjects, its role in this population was examined in a separate model.

All statistical analyses were conducted using Stata version 18 (StataCorp, College Station, TX). A two-tailed *p*-value below < 0.05 indicated conventional statistical significance.

## Results

### Patient characteristics

**Fig. 1:**
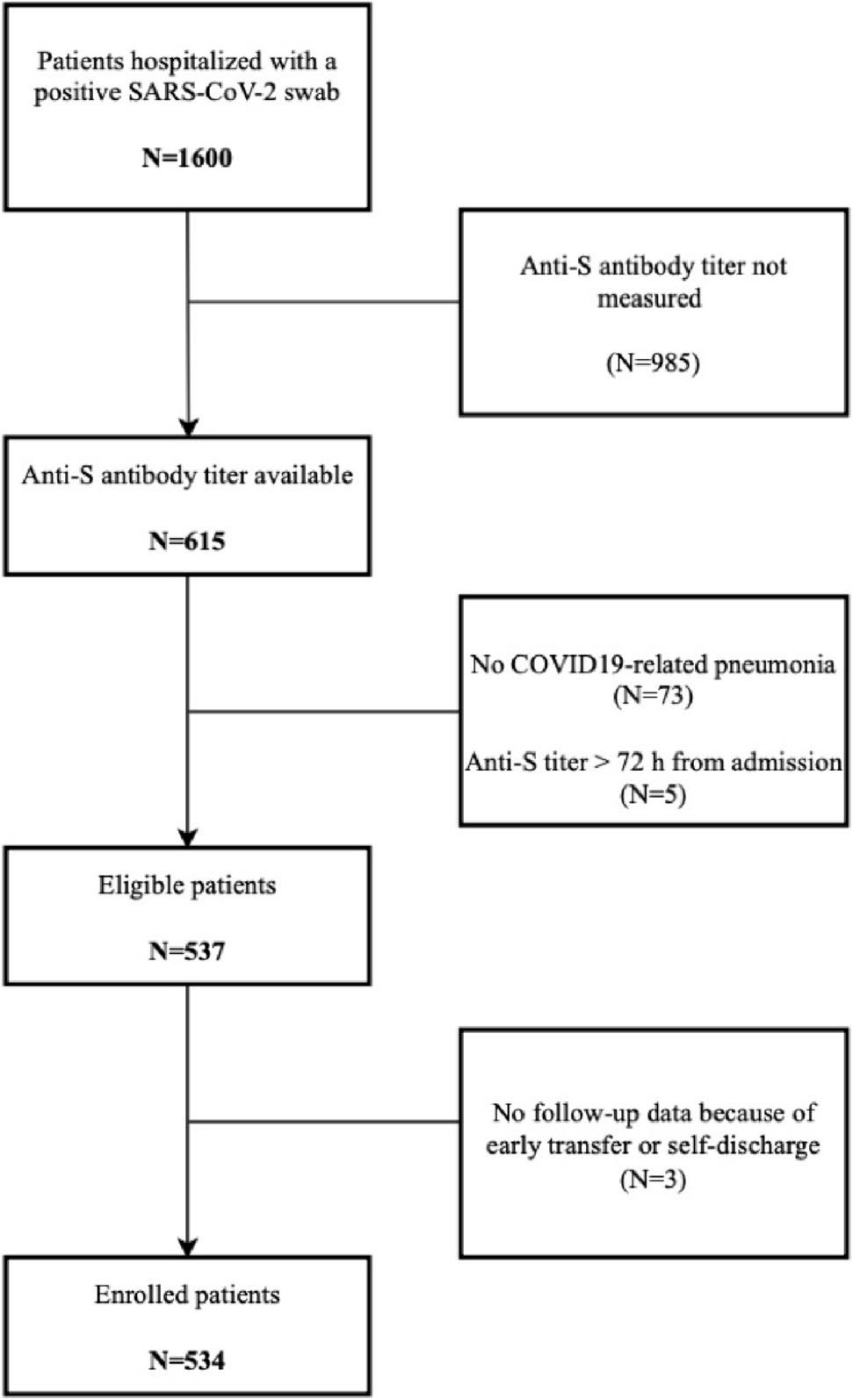
Patients’ disposition (CONSORT diagram)

A total of 1600 individuals were hospitalized with SARS-CoV-2 infection during the study period, 534 of whom met the eligibility criteria and were therefore enrolled in the study. The CONSORT diagram, depicting the process of eligibility assessment and the reasons for exclusion from the study, is shown in Fig. 1.

Patients enrolled were predominantly male (62.9%), with a mean age of 72 years; 61.4% of them had received at least 1 dose of COVID-19 vaccine while 42.5% had ≥ 1 immunosuppressive condition, the most common of which were diabetes (20.6%), use of immunosuppressive therapy (11.6%) and hematological malignancies (10.1%).

Median antibody titer in the overall cohort was 216 BAU/ml (IQR 11–2080). One hundred and four patients (19.5%) had undetectable levels (< 4.8 BAU/ml) of antibodies (9.4% among vaccinated; 35.4% among unvaccinated).

Vaccinated subjects were, on average, older (75 vs 65 years; *p* < 0.001) and had a higher probability of immunocompromised status. In particular, 53% of the vaccinated cohort had at least one immunosuppressive condition, in contrast to 25.7% in the unvaccinated cohort (*p* < 0.001). These and other baseline characteristics, overall and stratified by vaccine status, are detailed in Table 1.

**Table 1:** Patient characteristics in the overall, vaccinated and unvaccinated cohorts

Patient characteristics	Vaccinated	Unvaccinated (*N* = 206)	Overall (*N* = 534)
	(*N* = 328)		
Age**,** years—mean (SD)	75 (12)	65 (15.8)	71.5 (14.4)
Male gender, *N* (%)	209 (63.7)	127 (61.6)	336 (62.9)
Days from symptoms onset, *N* (%)			
≤5 days	159 (48.5)	47 (22.8)	206 (38.6)
6–10 days	108 (32.9)	89 (43.2)	197 (36.9)
>10 days	61 (18.6)	70 (34.0)	131 (24.5)
Oxygen support on admission day, *N* (%)			
No oxygen	16 (4.9)	10 (4.9)	26 (4.9)
Nasal prongs or facial mask with FiO_2_ < 50%	145 (44.2)	80 (38.8)	225 (42.1)
Facial mask with FiO_2_ ≥ 50%	112 (34.2)	73 (35.4)	185 (34.7)
Non-invasive ventilation (CPAP helmet)	50 (15.2)	43 (20.9)	93 (17.4)
Mechanical ventilation	5 (1.5)	0 (0)	5 (0.9)
Immunosuppressive conditions, *N* (%)			
Diabetes	78 (23.8)	32 (15.5)	110 (20.6)
Immunosuppressive therapy	53 (16.1)	9 (4.3)	62 (11.6)
Hematological malignancy	47 (14.3)	7 (3.4)	54 (10.1)
Solid malignancy	30 (9.1)	10 (4.8)	40 (7.5)
Renal failure (stage IV CKD or ESRD)	23 (7.0)	3 (1.5)	26 (4.9)
Organ transplant recipient	11 (3.3)	1 (0.5)	12 (2.2)
People living with HIV	1 (0.3)	1 (0.5)	2 (0.4)
Liver cirrhosis	1 (0.3)	0 (0)	1 (0.2)
At least 1 of the previous conditions, *N* (%)	174 (53.0)	53 (25.7)	227 (42.5)
Treatment with mAb, *N* (%)	16 (4.9)	55 (26.7)	71 (13.3)
Number of vaccine doses received, *N* (%)			
One dose	35 (10.7)	N.A	N.A
Two doses	180 (54.9)	N.A	N.A
Three doses	113 (34.4)	N.A	N.A
Time between last vaccine dose and hospital admission, days–median (IQR)	144 (77–200)	N.A	N.A
SARS-CoV-2 Variant, *N* (%)			
Delta	85 (25.9)	91 (44.2)	176 (33.0)
Omicron	72 (22.0)	23 (11.2)	95 (17.8)
Other	2 (0.6)	1 (0.5)	3 (0.6)
Unknown	169 (51.5)	91 (44.1)	260 (48.6)
Anti-S antibody level, BAU/ml—median (IQR)	1370 (116–2080)	15.5 (0–107)	216 (11–2080)
Undetectable anti-S level (< 5 BAU/ml), *N* (%)	31 (9.4)	73 (35.4)	104 (19.5)

*Anti-S* anti-spike, *BAU* binding antibody unit, *CKD* chronic kidney disease, *CPAP* continuous positive airway pressure, *ESRD* end-stage renal disease, *FiO*_*2*_ fraction of inspired oxygen, *HIV* human immunodeficiency virus, *mAb* monoclonal antibodies, *N.A* Not Applicable

### Factors associated with anti-spike antibody level

The median antibody titer was significantly higher among vaccinated than among unvaccinated patients (1370 vs 15.5 BAU/ml; Mann–Whitney *U* test *p* < 0.001).

Among vaccinated individuals, univariate quantile regression showed that a symptom duration exceeding 6 days was significantly associated with a higher median antibody level (versus ≤ 5 days, 6–10 days + 1352 BAU/ml [95%CI + 549, + 2155] *p* = 0.001; > 10 days + 1442 [95%CI + 72, + 2811] *p* = 0.039). Factors associated with a lower antibody titer, on the other hand, were the presence of hematological malignancies (−2064 BAU/ml [95%CI −2583, −1545] *p* < 0.001), the use of immunosuppressive drugs (−2037 BAU/ml [95%CI −2411, −1663] *p* < 0.001), and chronic kidney disease (−1363 BAU/ml, [95%CI −2743, + 17] *p* = 0.053). Age, gender, and the remaining immunosuppressive conditions were not associated with antibody titer to a significant extent. Using multivariable analysis, symptom duration was confirmed to be a predictor of antibody levels (versus ≤ 5 days, 6–10 days: +875 [95%CI +436, +1314] *p* < 0.001; > 10 days: +875, [95%CI +341, +1408] *p* = 0.001), while hematological malignancies (−985 BAU/ml [95%CI −1601, −368] *p* = 0.002) and use of immunosuppressive drugs (−859 BAU/ml [95%CI −1446, −272] *p* = 0.004) remained significantly and independently associated with a lower median antibody titer.

In the unvaccinated cohort, symptom duration was the only factor significantly associated with antibody level (+60 BAU/ml, [95%CI +16, +104] *p* = 0.008). Furthermore, renal failure was marginally associated with a lower median titer (−16 BAU/ml [95%CI −33, +1.2] *p* = 0.069). Neither demographic characteristics nor presence of immunosuppressive conditions resulted to be associated with antibody titer to a significant extent.

Using multivariable analysis, symptom duration was confirmed to be the only significant predictor of antibody titer (versus ≤ 5 days, 6–10 days: +2 [95%CI −31, +35] *p* = 0.0893; > 10 days: +55 [95%CI +22, +89] *p* = 0.001), independently of age, presence of severe renal impairment, or immunosuppressive conditions.

### Anti-spike antibody level and risk of death/mechanical ventilation

A total of 134 patients met the outcome of interest by day 30. Of these, 42 patients needed mechanical ventilation (13 of whom subsequently died), while 92 died without undergoing mechanical ventilation.

### Vaccinated population

**Fig. 2:**
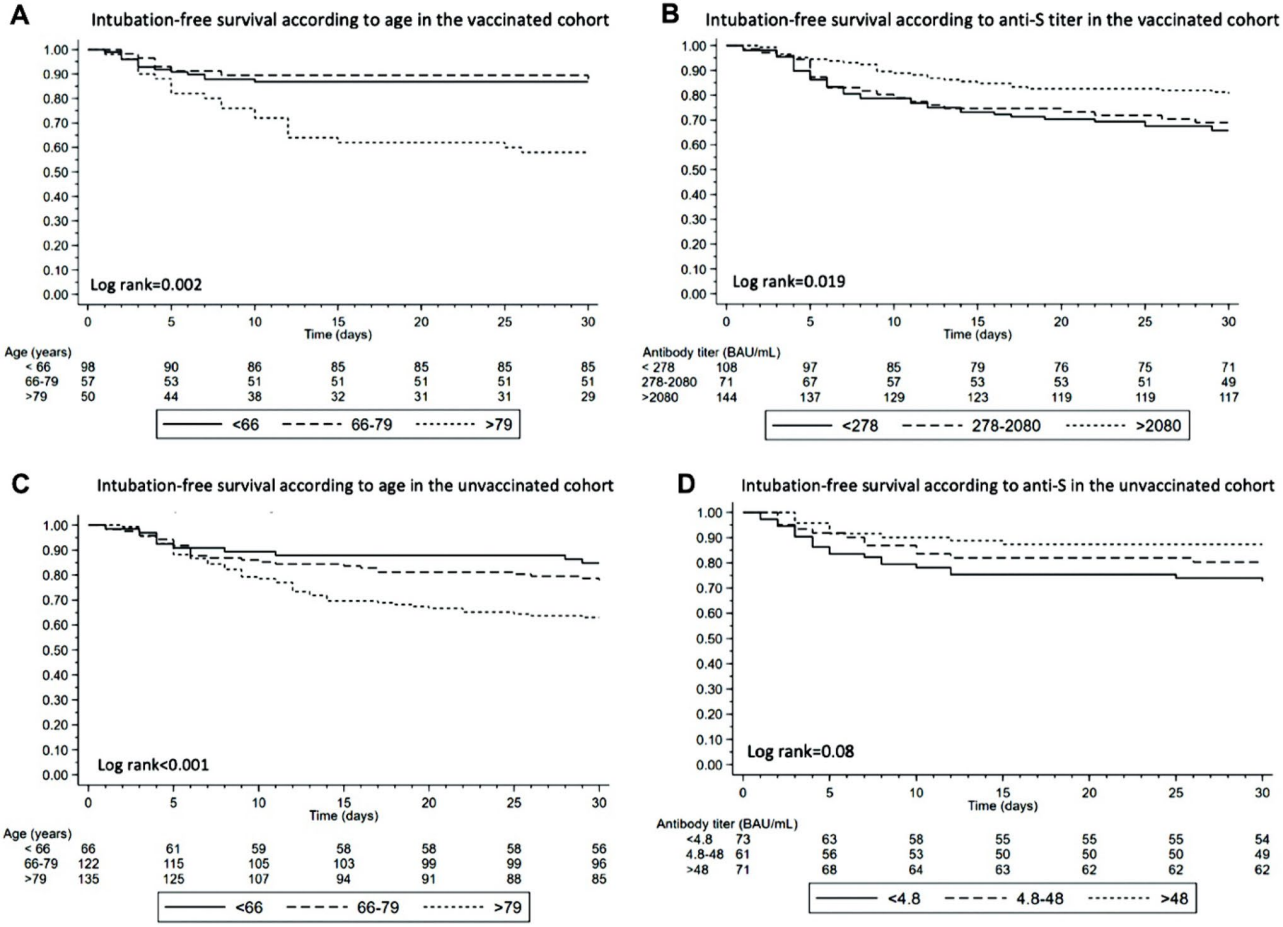
Kaplan–Meier curves for intubation-free survival according to age and anti-S titer in the vaccinated (**A**, **B**) and unvaccinated cohort (**C**, **D**)

Figure 2A , B shows the Kaplan–Meier estimated probability of remaining alive and free from ventilation stratified by age and anti-S titer, among vaccinated individuals. Increasing age (Fig. 2A, log-rank test *p* = 0.002) and lower anti-S levels (Fig. 2B, p = 0.019) were significantly associated with heightened mortality or the requirement for invasive mechanical ventilation.

Using univariate logistic regression analysis (Table 2), a significant association was found between anti-S titer and the risk of death or intubation within 30 days of hospitalization. Specifically, for each log_2_ increase in the anti-S level, there was a risk reduction of 10% (OR 0.90 [95%CI 0.83–0.97] *p* = 0.009). Furthermore, longer symptom duration was significantly associated with lower risk of death or intubation (*versus* ≤ 5 days, 6–10 days OR 0.75 [95%CI 0.44–1.29] *p* = 0.308; > 10 days OR 0.45 [95%CI 0.22–0.94] *p* = 0.034), while increasing age (per 1 year increase, OR 1.03 [95%CI 1.01–1.05] *p* = 0.004) and the presence of solid malignancies (OR 2.47 [95%CI 1.15–5.29] *p* = 0.020) were associated with a risk increase. Neither gender nor other investigated comorbidities demonstrated a statistically significant association with the outcome measure.

**Table 2:** Results from uni- and multivariable analysis assessing predictors of 30 days of death or intubation

Vaccinated individuals (*n* = 328)	Univariate analysis			Multivariable analysis		
	OR	95%CI	*p*	aOR	95%CI	*p*
Anti-S Ab titer (log_2_ BAU/ml)	0.9	0.84–0.97	0.009	0.89	0.81–0.97	0.009
Age (years)	1.03	1.01–1.05	0.004	1.04	1.01–1.06	0.003
Male gender	1.34	0.80–2.23	0.264	–	–	–
Days of symptoms						
≤5 days	1					
6–10 days	0.75	0.44–1.29	0.308	1.07	0.6–1.93	0.808
>10 days	0.45	0.22–0.94	0.034	0.5	0.24–1.08	0.08
Diabetes	1.01	0.57–1.78	0.972	–	–	–
Renal failure (stage IV CKD or ERSD)	1.13	0.45–2.85	0.792	–	–	–
Hematological malignancy	1.56	0.81–2.98	0.183	1.41	0.67–3	0.366
Solid malignancy	2.47	1.15–5.29	0.02	3.01	1.34–6.79	0.008
Immunosuppressive therapy	1.13	0.59–2.15	0.705	–		–
Transplant recipient	1.49	0.42–5.20	0.535	–	–	–

Unvaccinated individuals (*n* = 206)	Univariate analysis			Multivariable analysis		
	OR	95%CI	*p*	aOR	95%CI	*p*
Anti-S Ab titer (log_2_ BAU/ml)	0.89	0.78–1.03	0.129	0.86	0.73–1.01	0.064
Age (years)	1.05	1.02–1.07	< 0.001	1.04	1.01–1.07	0.004
Male gender	0.62	0.31–1.22	0.168	–	–	–
Days of symptoms						
≤5 days	1					
6–10 days	0.84	0.37–1.92	0.689	–	–	–
>10 days	0.49	0.19–1.24	0.131	–	–	–
Diabetes	3.42	1.52–7.69	0.003	3.05	1.24–7.48	0.015
Renal failure (stage IV CKD or ERSD)	1.97	0.17–22.3	0.582	–	–	–
Hematological malignancy	5.65	1.21–26.3	0.027	1.98	0.28–14.14	0.494
Solid malignancy	0.42	0.05–3.41	0.417	–	–	–
Immunosuppressive therapy	16.2	3.2–81.3	0.001	16.33	2.23–119.52	0.006

*aOR* adjusted odds ratio, *BAU* binding antibody unit, *CI* confidence interval, *CKD* chronic kidney disease, *ESRD* end-stage renal disease, *OR* odds ratio

In the final multivariable model, after adjustment for age, symptom duration, and presence of hematological or solid malignancies, increasing anti-S levels remained significantly associated with a lower risk of death or intubation (per log_2_ BAU/ml, OR 0.89 [95%CI 0.81–0.97] *p* = 0.009). Age (per year, OR 1.04 [95%CI 1.01–1.06] p = 0.003) and presence of solid malignancies (OR 3.01 [95%CI 1.34–6.79] p = 0.008) were also confirmed to be independent predictors of the outcome.

**Fig. 3:**
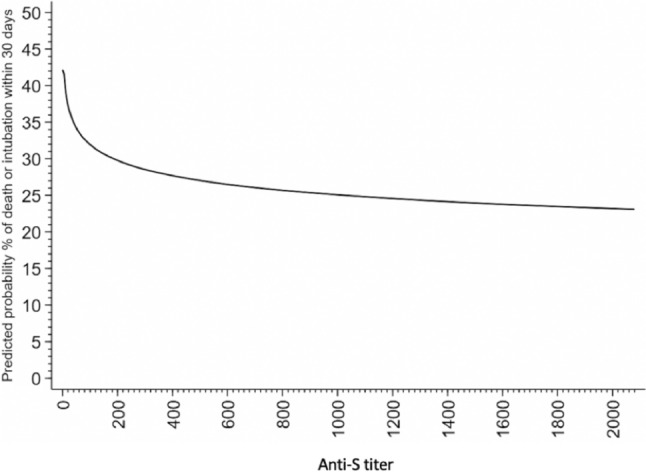
Predicted probability of clinical progression within 30 days as a function of the Anti-S levels according to the logistic regression model (vaccinated individuals)

Since establishing a distinct threshold of antibody level that distinguishes between survivors and non-survivors was not possible, owing to significant overlap in antibody titers, we graphically represented the probability of death or intubation predicted by our model, in relation to the observed antibody levels within the vaccinated cohort (Fig. 3). Through data extrapolation, we identified that an antibody level of 350 BAU/ml corresponded to the probability of death or need of mechanical ventilation observed in our population (28%), thus suggesting that antibody levels falling below this threshold may indicate a higher risk of adverse outcomes, such as death or intubation, compared to the general population.

### Unvaccinated population

As shown in Fig. 2C, D among unvaccinated individuals, those who were ≥ 80 years old had a significantly shorter time to death or mechanical ventilation than those who were younger (*p* = 0.001). Furthermore, antibody levels were marginally associated with the risk of death/intubation, although the difference did not reach statistical significance (*p* = 0.129). Using logistic regression analysis (Table 2), immunosuppressive therapy (OR 16.2 [95%CI 3.2–81.3] *p* = 0.001), diabetes (OR 3.42 [95%CI 1.52–7.69] *p* = 0.003), hematological malignancies (OR 5.65 [95%CI 1.21–26.3] * p* = 0.027), and age (OR 1.05 [95%CI 1.02–1.07] *p *< 0.001) were all significantly associated with the death or mechanical ventilation within 30 days of hospital admission. When the final multivariable model was constructed, adjusting for these possible confounders, anti-S Ab titer remained marginally associated with the outcome (per log2, OR 0.86 [95%CI 0.73–1.01] *p* = 0.064), independently of age (OR 1.04 [1.01–1.07] *p* = 0.004), immunosuppressive therapy (OR 16.33 [2.23–119.52] *p* = 0.006), and diabetes (OR 3.05 [1.24–7.48] *p* = 0.015).

In a separate multivariate model, we examined the net effect of anti-S antibody levels on the subsequent risk of clinical progression, after adjusting for the possible mitigating effect of mAb treatment. After accounting for mAb treatment, the association between higher antibody levels and reduced risk of progression further strengthened, achieving statistical significance (adjusted OR 0.81 [95%CI 0.68–0.96] *p* = 0.018). Of note, mAb treatment appeared to be associated with a lower risk of progression (adjusted OR 0.32 [95%CI 0.10–0.97] *p* = 0.045), independently of other possible confounders previously considered.

## Discussion

Understanding the factors that influence the antibody response against the SARS-CoV-2 virus and the clinical evolution of COVID-19 is important for both clinical practice and public health strategy planning. Moreover, evidence regarding the association between the extent of the humoral response, measured by anti-S antibody level, and the risk of clinical progression of COVID-19-related pneumonia is lacking and somehow conflicting [4, 6–8, 14, 22].

In our study, the factors associated with anti-S levels in patients with COVID-19 pneumonia varied depending on their vaccine status. Among individuals who had been vaccinated, the use of immunosuppressive therapies and the presence of hematological malignancies were associated with lower antibody titers, despite vaccination. This finding aligns with the existing literature, which has shown significantly lower seroconversion rates in patients with hematological neoplasms, particularly those involving the B cell lineage [23, 24], and in those on immunosuppressive drugs [25], especially anti-CD20 therapies [24, 26]. Additionally, a meta-analysis suggested that the immunogenicity induced by vaccination may vary significantly among patients receiving immunosuppressive therapy, potentially influenced by the number of drugs taken and the time since the last administration [26]. In contrast, advanced or end-stage chronic kidney disease, diabetes, and solid malignancies did not show, in our cohort, any significant association with low antibody titers upon hospitalization. Consistently with this finding, good probability of seroconversion after vaccination, although inferior to that of immunocompetent individuals, has been previously shown among patients with end-stage renal disease [27, 28], diabetes [29, 30], or solid malignancies [31, 32]. Of note, previous studies have also documented reduced antibody responses in patients receiving solid organ transplants [32] and those with HIV infections [33]. However, individuals with these characteristics were insufficiently represented in our sample, thereby preventing a thorough assessment of such associations.

Unvaccinated patients presented, as expected, lower antibody levels than the vaccinated individuals, and, among them, a higher number of patients had undetectable antibodies. In this group of patients, the only factor significantly associated with the magnitude of the antibody response was the duration of symptoms. This observation is likely attributable to the physiological dynamics of antibody production. Previous studies have documented that, on average, seroconversion occurs approximately 10 days after SARS-CoV-2 infection [34]. Notably, in the unvaccinated population included in our study, neither age nor immunodepression conditions were associated with early seroconversion or antibody level. Taken together, these data suggest that, in an unvaccinated population that has developed severe disease, at least in the initial phase of infection, the capacity to mount an antibody response does not appear to be influenced by comorbidities and immunosuppression.

Despite being conducted amidst and after the vaccination campaign roll-out, our study revealed considerably high overall mortality rates and elevated risk of disease progression to critical forms. In our cohort, approximately 20% of the patients died within 30 days and an additional 8% underwent mechanical ventilation due to respiratory failure. Such death rates do not significantly differ from those reported in the first COVID-19 waves [35]. Notably, in our study, mortality rates in the vaccinated and the unvaccinated population were comparable. While COVID-19-related mortality has been shown to decrease in parallel with vaccination roll-out and potential viral evolution toward reduced severity, it should be noted that our study enrolled individuals already affected with severe forms of the disease, manifesting clinical and/or radiological signs of pneumonia. Furthermore, those who developed COVID-19 pneumonia despite vaccination were likely to be the most vulnerable, predominantly older, and prone to severe comorbidities. These findings underscore that, even in the era of vaccination, COVID-19 still poses a life-threatening risk when pneumonia develops [16]. They also emphasize the possibility of vaccine failure, particularly in the elderly and immunocompromised individuals, who remain at an increased risk of unfavorable outcomes compared to the general population.

The primary aim of our study was to assess whether anti-S antibody level was associated with the risk of critical or fatal disease among patients with COVID-19 pneumonia. Additionally, we sought to determine the utility of this marker in identifying patients at an elevated risk of severe outcomes, independently of other important prognostic factors. In the vaccinated population, anti-S antibody titer was significantly associated with the likelihood of developing clinical outcomes. Specifically, a twofold increase in the antibody titer corresponded to a 10% reduction in the risk of death or intubation. This association held true even after adjusting for other potential confounders, such as patient age, symptom onset, and especially the presence of conditions causing immunosuppression. Similarly, also among those not vaccinated against COVID-19, higher antibody levels at the time of admission were associated with a lower probability of death or intubation, although the association did not reach statistical significance. Our results are in line with previous evidence obtained in the early phases of the pandemic and suggesting that low or undetectable levels of anti-S or neutralizing antibodies measured at the time of hospitalization can predict an unfavorable outcome, while the development of specific antibody response may coincide with a favorable disease progression [5, 7, 36, 37]. All these studies, however, have been conducted mainly on unvaccinated individuals. Our findings confirm and expand this evidence, suggesting that antibody levels upon admission have significant prognostic value in predicting the subsequent risk of disease progression also (and particularly) among individuals who have already been vaccinated. Notably, we observed not only a qualitative association but also a quantitative one, suggesting that higher antibody levels are associated with better outcomes. This quantitative relationship could have potential clinical implications, such as guiding the monitoring of antibody titers in patients and aiding in the selection of individuals who may benefit from more aggressive treatment approaches. For instance, patients with persistently low antibody levels may necessitate more vigilant observation and an early use of antiviral medications or monoclonal antibodies. Measuring antibody levels could also be useful in stratifying the risk of disease progression, so that individuals at higher risk of severe disease could receive prompt and intensive care. It should be noted, however, that, given the inherent differences between vaccinated and unvaccinated individuals, the analyses of antibody titers may hold distinct implications for each group. In the vaccinated group, the assessment of antibody titers provides an indicator of the rapid recall of the immunity originally induced by the vaccine. This dynamic reflects the accelerated and enduring protection conferred by the vaccination, thereby serving as a surrogate measure of vaccine efficacy. Meanwhile, in the unvaccinated group, antibody titers reflect the onset of the specific immune response, the extent of which could be impacted by the overall health of the immune system. These differences translate in varying levels of antibodies upon admission, diverse extents of the association between antibody levels and outcomes, and distinct thresholds that predict a worse outcome.

In our cohort, identifying a precise anti-S threshold to differentiate survivors from patients who died or developed critical respiratory failure proved challenging, due to significant overlap in the antibody titers in the two populations. Nevertheless, we have proposed a critical value of 350 UI/ml among vaccinated subjects, below which, as per our model, the predicted risk of an unfavorable outcome was higher compared to the rest of the population. However, the prognostic validity of this cut-off needs to be prospectively tested in an independent population.

Although beyond the scope of our study, the use of monoclonal antibodies was found to be associated with a lower risk of clinical progression and death in the non-vaccinated population. In addition, in an analysis accounting for the possible mediation effect of mAb administration, the association between anti-S antibody titer and risk of clinical progression strengthened, thus suggesting that treatment with mAb can mitigate the risk of clinical progression in patients with very low or undetectable antibody titers. These findings are consistent with previous results of randomized controlled trials [38, 39], and suggest that measuring anti-S antibody titers could help to identify the niche of patients who may benefit from mAb treatment in the hospitalization setting. The result should, however, be interpreted cautiously, because we cannot exclude a preferential use of monoclonal antibodies among patients with less advanced disease and with a more favorable prognosis, as perceived by the treating physicians. Indeed, according to Italian prescribing criteria, patients with severe respiratory failure requiring non-invasive ventilation were ineligible for monoclonal antibody treatment.

Our study has some limitations that must be acknowledged. First, given its observational nature, residual confounding due to unmeasured factors cannot be excluded, despite partly mitigated by conducting a multivariate regression analysis. Although steroid and antiviral treatments were prescribed to most patients with COVID-19-related pneumonia, we did not collect detailed information on these treatments. However, we do not believe that this could have affected our analysis, as there is no reason to think that a relationship between their use and antibody levels, which were the focus of our study, exists. Second, the sample was non-randomly selected and based on the availability of antibody titer measurement during hospitalization (convenience sample). This inclusion mode in the study may limit the generalizability of the results. Third, we didn’t address the potential influence of the ongoing evolution of the pandemic, which includes changes in both population immunity and viral characteristics. Specifically, our study did not account for the potential impact of the emergence of new viral variants (notably, the Omicron variant) during the patient inclusion period. Furthermore, the information regarding previous COVID-19 infections within our cohort is inconsistent. Although all patients in our study were admitted to the hospital for the first time due to COVID-related pneumonia, some of them might have had asymptomatic or mild infections in the past. Despite the complexities that arise from the pandemic dynamics, we believe that our findings offer valuable insights into predicting the outcomes of severe COVID-19 cases, regardless of whether the patient has vaccine-induced or hybrid immunity. It is important to note that we currently have no reason to doubt that the association between antibody levels and protection from fatal or critical outcomes would apply in a population with hybrid immunity or with different viral variant dominance. However, further confirmation is needed through ongoing research. Eventually, it is important to note that although anti-S antibody levels correlate excellently with neutralizing antibody response, neutralization assays against circulating variants may better reflect immune system protection. Nevertheless, the choice to use a commercially available assay that quantifies the anti-S antibody response responds to the need for a reproducible, validated, and easily executable test in all contexts, for clinical purposes.

In conclusion, immunosuppressive therapies and severe comorbidities may hamper humoral response directed against SARS-CoV-2, particularly among vaccinated individuals. Regardless, anti-S levels measured upon hospital admission seem convincingly associated with the risk of death or intubation among hospitalized patients with COVID-19 pneumonia, both in vaccinated and unvaccinated individuals. Further studies are warranted to investigate the potential clinical applications of monitoring antibody titers, particularly in immunocompromised patients, with a focus on elucidating the role of antibodies as prognostic markers.

## Data Availability

No datasets were generated or analysed during the current study.
